# The GuideLiner Catheter: A Useful Tool in the Armamentarium of the Interventional Cardiologist

**Published:** 2015-10-27

**Authors:** Marouane Boukhris, Salvatore Azzarelli, Salvatore Davide Tomasello, Zied Ibn Elhadj, Francesco Marzà, Alfredo R. Galassi

**Affiliations:** 1 *Cannizzaro Hospital, University of Catania, Catania, Italy.*; 2 *Faculty of Medicine of Tunis, University of Tunis El Manar, Tunisia.*

**Keywords:** *Percutaneous coronary intervention*, *Cardiac catheters*, *Equipment and supplies*

## Abstract

Regardless of the clinical setting, a good back-up represents one of the most important conditions to ensure guide wire and balloon advancement and stent delivery. As a “mother and child” system, the GuideLiner catheter (Vascular Solutions Inc., Minneapolis, MN, USA) provides an extension to the guide catheter with better coaxial alignment and stability. We report two didactic cases showing the usefulness of the GuideLiner device in everyday catheterization laboratory practice. The first case was a primary percutaneous coronary intervention (PCI) in a 71-year-old diabetic man admitted for inferior ST-elevation myocardial infarction, related to tight proximal stenosis in a dominant tortuous and calcified left circumflex. The second case was an elective PCI in a 76-year-old man admitted for stable angina (Canadian Cardiovascular Society [CCS] class III), related to focal intra-stent restenosis of a saphenous venous graft to the left anterior descending. In both cases, the GuideLiner catheter provided a good back-up insuring the success of PCI and drug-eluting stents implantation, with a good in-hospital outcome.

## Introduction

Thanks to the development of equipment and techniques,^1^^, ^^2^ particularly in the wake of chronic total occlusion revascularization,^3^^-^^5^ percutaneous coronary intervention (PCI) has reduced the surgical indications of myocardial revascularization. Regardless of the clinical setting, a good back-up represents one of the most important conditions to ensure guide wire and balloon advancement and stent delivery, thereby leading to PCI success.^6^ The use of stiffer guide wire, anchoring balloon technique, and deeper intubation of the guide catheter may represent, in some cases, reliable options able to improve back-up support.^7^^-^^9^ As a “mother and child” system, the GuideLiner catheter (Vascular Solutions Inc., Minneapolis, MN, USA) provides an extension to the guide catheter with a better coaxial alignment and stability. We report two didactic cases showing the usefulness of the GuideLiner device in everyday catheterization laboratory practice.

**Figure 1:**
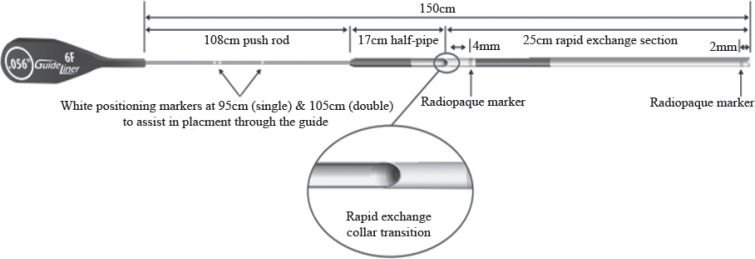
GuideLiner V3 Catheter (Vascular Solutions Inc., Minneapolis, MN, USA) (www.vasc.com)

The GuideLiner catheter (Vascular Solutions Inc., Minneapolis, MN, USA) is a coaxial “mother and child” catheter mounted on a monorail system extending the guide catheter. It consists of a coaxial exchange system joined to a 125-mm compact metal hypotube, and a flexible extension of 20 cm with a radiopaque marker located 2.7 mm from the tip and an inner diameter 1-Fr size smaller than the guiding catheter. The GuideLiner V2 catheter provides additional extension of 5 cm and presents all polymer collar for increased flexibility. The more recent GuideLiner V3 catheter has a 25-cm cylinder at the distal tip and a half-pipe design facilitating smooth device entry and delivery (Figure 1). It is available in 4 sizes: 5.5, 6, 7, and 8 Fr.

Once the guide catheter and guide wire are placed, the GuideLiner catheter can be advanced over the guide wire through the hemostatic valve as an extension to the guide catheter. Subsequently, the procedure can be continued as usual, without need for disconnection and reattachment.

## Case Report # 1

**Figure 2:**
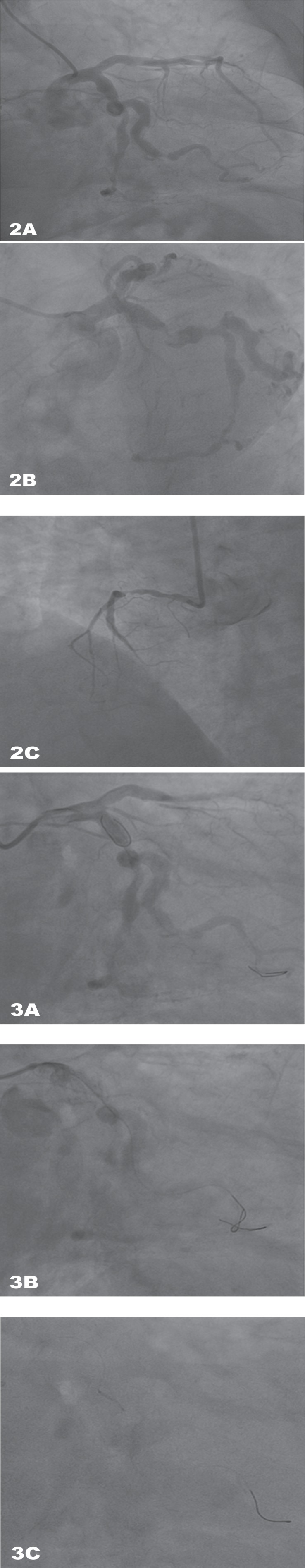
A and B) Right caudal and left caudal coronary angiography views, respectively, showing dominant tortuous and calcified left circumflex artery (LCx) with a tight proximal stenosis, followed by aneurysmal dilation and thrombus. The distal LCx was diseased with aneurysmal dilation, and the middle segment of the first obtuse marginal presented a tight stenosis. The left anterior descending was atheromatous but did not show any significant lesion. C) Left view, showing a minor diseased right coronary artery

**Figure 3:**
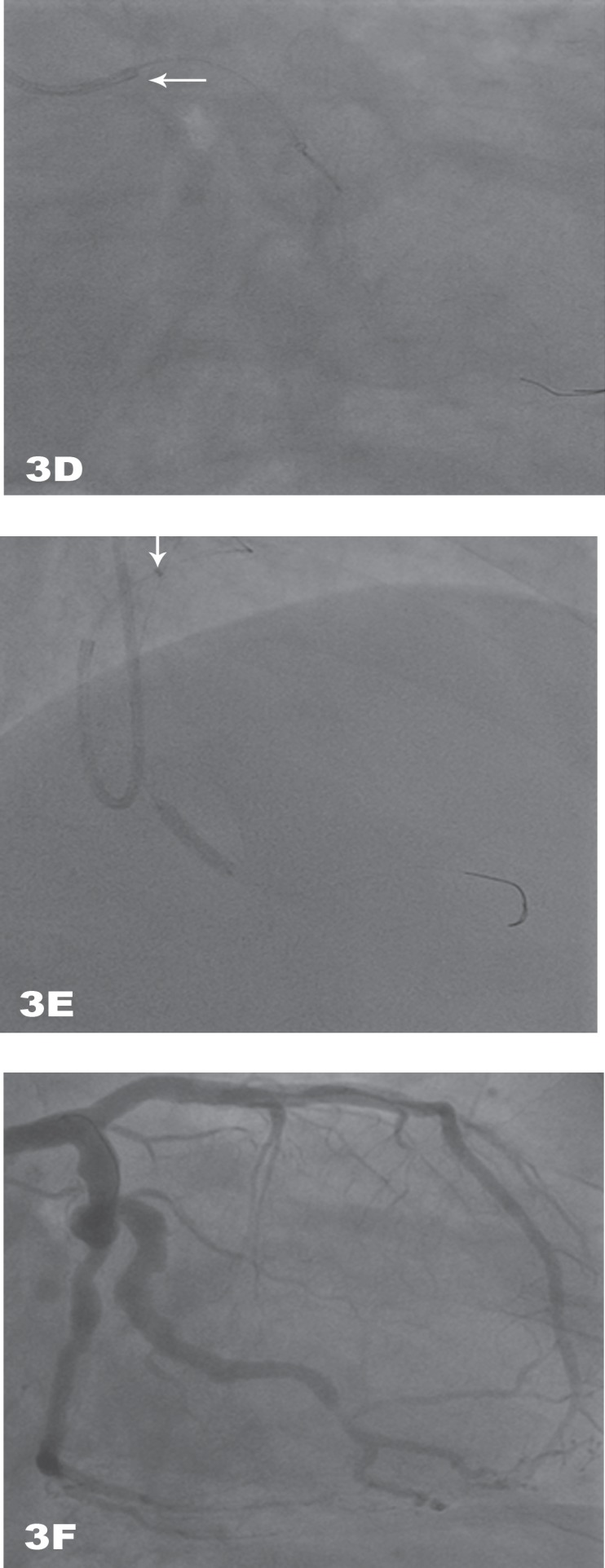
Right caudal coronary angiography views, showing A) second runthrough guide wire (Terumo, Japan) proving impossible to be advanced to the distal left circumflex artery (LCx). B) Two runthrough guide wires (Terumo, Japan) in the obtuse marginal (OM) to improve the support. C) Deinsertion of the guiding catheter from the left main due to balloon pre-dilation. D) Delivery of the stent to the middle segment of the OM through the GuideLiner V2 catheter (Vascular Solutions Inc., Minneapolis, MN, USA). E) Runthrough guide wire (Terumo, Japan) in the distal LCx and the delivery of three stents. F) Good final result.

A 71-year-old diabetic man was admitted for inferior ST-elevation myocardial infarction, 2 hours after chest pain onset. The patient received an intravenous bolus of aspirin (250 mg), loading dose of ticagrelor (180 mg), and primary PCI was indicated. Coronary angiography through the right radial access was performed, showing a dominant tortuous and calcified left circumflex (LCx) with a tight proximal stenosis, followed by aneurysmal dilation and thrombus. Additionally, the distal LCx was diseased with aneurysmal dilation, and the middle segment of the first obtuse marginal (OM) presented a tight stenosis. The left anterior descending (LAD) did not show any significant lesion, while the right coronary artery (RCA) was a minor diseased vessel (Figure 2). Because of the right subclavian tortuosity and poor back-up, a switch to 6-Fr right femoral access was decided. Intravenous Bivalirudin was administered. A 6-Fr EBU 3.5 (Cordis Co., USA) was engaged in the left main. A runthrough guide wire (Terumo, Japan) was laboriously advanced into the first OM, while a second wire proved impossible to be advanced to the distal LCx (Figure 3A). Laborious thromboaspiration was performed. The back-up was poor despite the two guide wires into the OM (Figure 3B), and only non-selective injections were obtained. Balloon predilation was responsible for the deinsertion of the guide catheter from the left main, and stent advancement was not possible (Figure 3C). At this moment, it was decided to place a “5-in-6” Fr GuideLiner V2 catheter (Vascular Solutions Inc., Minneapolis, MN, USA) in order to improve the support. The latter was carefully inserted into the guide catheter and advanced to the OM, allowing the delivery of the stent (3.5 × 12 mm) to its middle segment (Figure 3D). Then, the Runthrough guide wire (Terumo, Japan) was able to reach the distal LCx. The GuideLiner V2 catheter (Vascular Solutions Inc., Minneapolis, MN, USA) permitted the delivery of three stents in the distal LCx. Regarding the proximal aneurysm, the decision to implant a self-expanding stent was taken, and the GuideLiner V2 catheter (Vascular Solutions Inc., Minneapolis, MN, USA) was removed. A Stentys (Stentys SA, Paris, France) stent (3.5-4.5 × 22 mm) was implanted with a good final angiographic result (Figure 3F). The in-hospital stay was uneventful, and the patient was discharged after 3 days from the procedure on dual anti-platelet (ticagrelor and aspirin) therapy for 12 months.

The arrows show the distal radiopaque marker of the GuideLiner V2 catheter.

***Case Report # 2***

**Figure 4:**
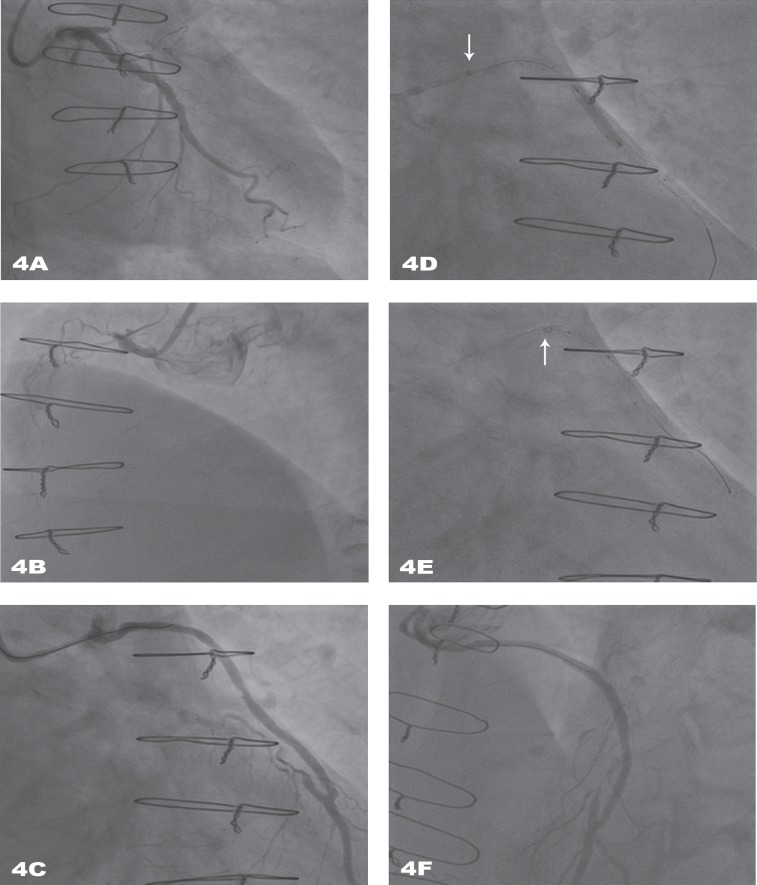
A) Caudal coronary angiography view, showing chronic total occlusion of the proximal left anterior descending (LAD) and atheromatous left circumflex (LCx) without significant stenosis. B) Left view, showing chronic total occlusion of the right coronary artery. C) Right cranial view, showing a tight intra-stent restenosis of the middle segment of the LAD saphenous graft. D) Coaxial alignment with the GuideLiner V2 catheter (Vascular Solutions Inc., Minneapolis, MN, USA) allowing the advancement of the balloon for predilation. E) Stent delivery through the GuideLiner V2 catheter to the middle segment of the LAD saphenous graft. F) Good final result.

A 76-year-old man was admitted for stable angina (CCS class III). Twenty years earlier, he had undergone coronary artery bypass graft intervention and received two saphenous grafts on the LAD and RCA. One year prior to his referral to us, a coronary angiography was performed for unstable angina, showing the occlusion of the saphenous graft RCA and a long stenosis in that of the LAD; thus, PCI of this latter bypass was performed using three stents, which conferred improvement of symptoms. Recent scintigraphy revealed ischemia of the anterior wall and absence of viability in the RCA territory. Coronary angiography via the left radial access, showed the chronic total occlusion of both LAD and RCA (native coronaries) and focal intra-stent restenosis of the LAD saphenous graft (Figure 4C). A bolus of Heparin 5000 UI was administered. A Judkins right 4 6-Fr guide catheter (Cordis Co., USA) was not able to appropriately cannulate the LAD saphenous graft (Figure 4D). Instead of exchanging the guide catheter, it was decided to employ a GuideLiner V2 catheter (Vascular Solutions Inc., Minneapolis, MN, USA) “5-in-6” Fr, to obtain coaxial alignment. The latter was carefully introduced into the graft, and it substantially improved the intubation. Additionally, a Runthrough guide wire (Terumo, Japan) was inserted. Pre-dilation with a 2.5 ×15 mm balloon was performed (Figure 3E), and then a 3.0 × 15 mm Everolimus-eluting stent was delivered to the middle segment of the saphenous graft (Figure 4E). Finally, post-dilation was performed using a non-compliant balloon (3.5 × 12 mm) with a good final angiographic result (Figure 4F). In-hospital stay was uneventful, and the patient was discharged after 4 days on dual anti-platelet therapy (aspirin and clopidogrel) for 12 months. 

The arrows show the distal radiopaque marker of the GuideLiner V2 catheter.

## Discussion

A good back-up of the guide catheter is crucial to advance guide wires and balloons and to deliver stents. The support can be improved by a deeper intubation of the guide catheter and the use of a buddy wire, stiffer guide wires, or anchoring balloons.^7^^-^^9^

 As a “mother and child” system, the GuideLiner enables deeper intubation. Indeed, as was described in the first case, it provides enhancement of the equipment delivery in challenging situations such as coronary tortuosity, severe calcifications, and coronary aneurysms. Furthermore, in patients with previous coronary artery bypass graft, the GuideLiner is recognized to be helpful in graft cannulation and enhancing guiding stability, as was the case in our second patient, for whom the GuideLiner use was preferred to exchanging the guide catheter. Other indications have also been described: the GuideLiner could be employed to perform selective contrast injections for a better target coronary vessel visualization with a smaller amount of contrast in chronic kidney disease patients,^10^ and might also be used as an aspiration device.^11^

In the Twente GuideLiner Registry,^12^ 70 lesions were treated through a “5-in-6” GuideLiner catheter. Among them, 97% were type B2/C (according to the American Heart Association [AHA] / American College of Cardiology [ACC] classification), 53% distal, and 23% showed heavy calcifications. The indications of the use of this “mother and child” device were as follows: to increase back-up and facilitate delivery (59%), achievement of coaxial alignment of the guide catheter (28%), and selective contrast injections (13%). The success rate reached in the latter study was as high as 93%,^12^ confirming other previous reports.^13^^-^^15^

The use of the GuideLiner proved generally safe; however, some complications have been documented. Murphy et al.^16^ reported a case of balloon damage at the site of metallic collar. Similarly, Seto and Kern^17^ witnessed the destruction of two stents upon attempted movement through the proximal GuideLiner collar. The new design of GuideLiner V3 aimed to prevent stent damage and dislodgement. On the other hand, De Man et al.^12^ encountered minor complications: one case of air embolism due to insufficient venting, and one case of stent dislodgement. Luna et al^15^ reported high number of pressure dampening cases during “6-in-7” Fr GuideLiner engagement. 

For the optimal use of this device, some recommendations should be respected. Time should be taken to vent the system in order to diminish the risk of air embolism. The GuideLiner should be inserted using a guide catheter over a first guide wire, in such a way that the tip protrudes a maximum of 10 cm. The connection to the flexible segment should be made in the straight portion of the guide catheter in order to facilitate the passage of devices along it. The operator should be sure that the proximal segment of the target coronary vessel is suitable for intubation. Indeed, if the lesion extends to the proximal part or if there is a sharp angulation, the extension system is not recommended. The model “5-in-6” Fr, employed in our cases, allows the passage of regular balloon catheters, contemporary optical coherence tomography catheters, and stents up to a nominal diameter of 3.5 mm. However, it does not permit the use of larger devices such as thrombectomy catheters, self-expanding stents, some intravascular ultrasound probes,^18^ and simultaneous kissing balloon inflations. 

Three other guide-catheter extensions are available: Guidezilla (Boston Scientific, Natick, MA, US) with hydrophilic coating and only available in a 6-Fr size; Heartrail (Terumo Corp., Tokyo, Japan) available in 5, 6, and, 7-Fr sizes; and Guidion (IMDS, Roden, Netherlands) available in 5, 6, 7, and 8-Fr sizes. 

## Conclusion

The guide catheter extension system is a useful tool to have in catheterization laboratory and a reliable alternative able to facilitate some difficult angioplasties. In case of the failure of “traditional tips and tricks” to improve the back-up in challenging cases, it can be employed as a “bail-out strategy”. However, with the growing experience with such a device, it can also be used as a first strategy to face anatomical difficulties. 

## Notes:

***This paper should be cited as:*** Boukhris M, Azzarelli S, Tomasello SD, Elhadj ZI, Marzà F, Galassi AR. The GuideLiner Catheter: a Useful Tool in the Armamentarium of the Interventional Cardiologist. J Teh Univ Heart Ctr 2015;10(4):208-214.
